# Does physiological-based cord clamping improve cerebral tissue oxygenation and perfusion in healthy term neonates? – A randomized controlled trial

**DOI:** 10.3389/fped.2022.1005947

**Published:** 2023-01-09

**Authors:** Bernhard Schwaberger, Mirjam Ribitsch, Gerhard Pichler, Marlies Krainer, Alexander Avian, Nariae Baik-Schneditz, Evelyn Ziehenberger, Lukas Peter Mileder, Johann Martensen, Christian Mattersberger, Christina Helene Wolfsberger, Berndt Urlesberger

**Affiliations:** ^1^ Research Unit for Cerebral Development and Oximetry, Medical University of Graz, Graz, Austria; ^2^ Research Unit for Neonatal Micro- and Macrocirculation, Medical University of Graz, Graz, Austria; ^3^ Division of Neonatology, Department of Pediatrics and Adolescent Medicine, Medical University of Graz, Graz, Austria; ^4^ Pediatric Intensive Care Unit, Division of General Pediatrics, Department of Pediatrics and Adolescent Medicine, Medical University Graz, Graz, Austria; ^5^ Institute for Medical Informatics, Statistics and Documentation, Medical University of Graz, Graz, Austria

**Keywords:** timing of cord clamping, neonatal transition, cerebral tissue oxygenation (index), cerebral blood volume (CBV), newborn infant, near-infrared spectroscopy, physiological-based cord clamping

## Abstract

**Objectives:**

To evaluate cerebral tissue oxygenation index (cTOI) during neonatal transition in a group of healthy full-term neonates receiving either a physiological-based approach of deferred cord clamping (CC) after the onset of stable regular breathing (PBCC group) or a standard approach of time-based CC < 1 min (control group). Secondary aim was to evaluate changes in cerebral blood volume (ΔCBV), peripheral arterial oxygen saturation (SpO2) and heart rate (HR) in those neonates.

**Materials and Methods:**

We conducted a randomized controlled trial (clinicaltrials.gov: NCT02763436) including vaginally delivered healthy full-term neonates. Continuous measurements of cTOI and ΔCBV using near-infrared spectroscopy, and of SpO2 and HR using pulse oximetry were performed within the first 15 min after birth. Data of each minute of the PBCC group were compared to those of the control group.

**Results:**

A total of 71 full-term neonates (PBCC: *n* = 35, control: *n* = 36) with a mean (SD) gestational age of 40.0 (1.0) weeks and a birth weight of 3,479 (424) grams were included. Median (IQR) time of CC was 275 (197–345) seconds and 58 (35–86) seconds in the PBCC and control group, respectively (*p* < 0.001). There were no significant differences between the two groups regarding cTOI (*p* = 0.319), ΔCBV (*p* = 0.814), SpO2 (*p* = 0.322) and HR (*p* = 0.878) during the first 15 min after birth.

**Conclusion:**

There were no significant differences in the course of cTOI as well as ΔCBV, SpO2 and HR during the first 15 min after birth in a group of healthy full-term neonates, who received either deferred CC after the onset of stable regular breathing or standard CC < 1 min. Thus, deferring CC ≥ 1 min following a physiological-based approach offers no benefits regarding cerebral tissue oxygenation and perfusion after uncomplicated vaginal delivery compared to a time-based CC approach.

## Introduction

Clamping of the umbilical cord is a major intervention after birth, causing the separation of the neonate from the placenta and the subsequent switch of gas exchange to the lungs. Although umbilical cord clamping (CC) is a quick and simple intervention, the timing of CC may have a large impact during the immediate neonatal transition period (1). Therefore, recent research focused on the appropriate timing of CC, distinguishing early (immediate) cord clamping (ECC) from deferred (delayed) cord clamping (DCC) without offering consensual definitions of these terms (1, 2).

The procedure of ECC has been the accepted standard of care for decades and was originally introduced as a package of care aiming for a reduction in maternal postpartum hemorrhage (3, 4). However, if CC is deferred, the blood flow between the placenta and the neonate may continue up to five minutes, potentially leading to a net transfer of blood from the placenta to the neonate known as “placental transfusion” (5–7). Placental transfusion has been shown to increase hemoglobin levels and cardiac output resulting in improved oxygen delivery in the newborn (1, 8).

Recently, animal experimental studies have shown that CC in the absence of pulmonary ventilation severely limits cardiac venous return, thus leading to fluctuations in blood pressure, hypoxia and restricted cardiac function (3). Once the neonate commences breathing, aeration of the lung triggers an increase in pulmonary blood flow and a decrease in pulmonary blood pressure, replacing umbilical venous return as the source of preload for the left ventricle (9). Therefore, “physiological-based cord clamping” (PBCC) was introduced offering the neonate time to establish spontaneous breathing while still being placentally supported (9). Whereas DCC is time-based (e.g., >1 min), PBCC is defined as clamping the umbilical cord after aeration of the lungs and establishment of pulmonary blood flow (3). In animal studies PBCC was associated with more stable heart rate, right ventricular output and carotid arterial blood flow, and increased pulmonary blood flow (10). Moreover, peripheral and cerebral tissue oxygenation and perfusion were more stable when CC was performed after the onset of ventilation (11).

However, available human data regarding PBCC are limited. It has been shown that performing PBCC in neonates is feasible (12–14). The stabilization of very preterm neonates receiving PBCC has been at least as effective as DCC (15), and may improve outcome at discharge for neonates born <32 weeks of gestation compared with ECC (16). Very recently, it has been demonstrated that in neonates ≥32 + 0 weeks of gestation requiring resuscitation at birth PBCC does not provide additional benefit over ECC in terms of heart rate (HR) and peripheral arterial oxygen saturation (SpO2) (17). In contrast, a Nepalese study found improved SpO2 and higher Apgar score in depressed term neonates after PBCC vs. ECC (18). Contradictory findings in human neonates might be due to different study populations (e.g., gestational age, vigorous vs. depressed neonates), different respiratory status after birth (spontaneous breathing vs. positive pressure ventilation to establish lung aeration) and different approaches of PBCC (different definition or methods to evaluate lung aeration).

The primary aim of the present study was to investigate whether a physiological-based approach of deferred cord clamping (CC) after the onset of stable regular breathing (PBCC group) compared to a standard approach of time-based CC < 1 min (control group) resulted in differences in cerebral tissue oxygenation index (cTOI) in vaginally delivered healthy term neonates. Secondary aim was to investigate changes in cerebral blood volume (ΔCBV), SpO2 and HR in those neonates. Following the publication of Smit et al. (19), we hypothesized that there would be higher SpO2 and lower HR in the PBCC group. Additionally, according to animal data (10, 11), we expected a smaller drop in cardiac output after clamping the cord in the PBCC group. Therefore, we hypothesized that cTOI would be higher in the PBCC compared to the control group. In case of increased cTOI values (representing an increase in cerebral oxygen delivery), we hypothesized that there would consecutively be a more pronounced CBV decrease in the PBCC group.

## Materials and methods

### Design

This randomized controlled trial was conducted at the Division of Neonatology of the Medical University of Graz (Austria, Europe). Between September 2016 and August 2019 full-term neonates were included in this study, provided written informed consent had been obtained from the parents prior to birth. The Regional Committee on Biomedical Research Ethics of the Medical University of Graz approved the study protocol (No. 28-087 ex 15/16). This trial was registered at ClinicalTrials.gov (Identifier: NCT02763436). Reporting of the study conforms to Consolidated Standards of Reporting Trials CONSORT 2010 statement (20).

### Participants

Vaginally delivered healthy full-term neonates (37 + 0 to 41 + 6 weeks of gestation) in vertex position were included, whenever research fellows and equipment were available and informed consent had been obtained prior to birth. Predefined exclusion criteria were maternal peripartum complications, presence of neonatal congenital malformations and requirement of respiratory support within 15 min after birth resulting in immediate cord clamping and transfer to the resuscitation table. Respiratory support was initiated in case of HR < 100 beats per minute and/or pronounced signs of respiratory distress (grunting, tachypnea and increased work of breathing). The exclusion of neonates requiring respiratory support was necessary due to the fact that the study equipment couldn't be moved to the resuscitation table within the required time.

### Intervention

PBCC group (intervention): The neonate was placed on mother's chest/abdomen with intact umbilical cord. Tactile stimulation was provided to the neonates. The cord was clamped after onset of stable regular breathing (assessed by the attending neonatologist), which was defined as a regular breathing pattern with a respiratory rate of at least 30 per minute and the absence of signs of respiratory distress such as grunting, tachypnea and increased work of breathing. We expected CC times for this physiological-based approach to be 2 to 4 min after birth.

Control group: The standard of CC at our center at the time of study initiation was a time-based approach, in which the umbilical cord was clamped approximately 30–40 s after birth. Prior to CC the neonate was positioned between the thighs of the mother and tactile stimulation was provided to the neonates. After CC the neonate was placed on the mother's chest/abdomen.

### Study protocol

Immediately after delivery of the neonate's head, the near-infrared spectroscopy (NIRS) sensor was placed on the left forehead. After complete delivery of the neonate, a pulse oximeter sensor was additionally fixed on the right palm or wrist to monitor pre-ductal SpO2 and HR.

A stopwatch was started at birth (after complete delivery of the neonate). The time points of CC, the first displayed NIRS values, the first displayed SpO2 values, and the initiation of skin-to-skin contact on the mother's chest/abdomen, were noted. Continuous measurements were performed during the first 15 min after delivery. Umbilical artery blood was drawn from a double-clamped cord segment and analyzed within 15 min. Additionally, we planned to perform an echocardiography between 15 and 30 min after birth in each patient.

### Outcome parameters and sample size calculation

The primary outcome parameter of the present study was cTOI. Polglase et al. studied lambs after Caesarean section (11). In the group of lambs with immediate CC, they observed a decrease of cTOI, contrary to the PBCC group, in which higher cTOI values were observed. In a previous study in neonates after vaginal delivery, cTOI values of 49% (±22%) were observed 3 min after delivery with ECC (21). Assuming a cTOI difference of 15% being clinically significant, the effect of PBCC should result in cTOI values of at least 64% at 3 min. To detect the described difference using a *t*-test with a power of 80%, 35 neonates have to be included in each group. Assuming a dropout rate of 10%, we calculated a sample size of 78 neonates.

Secondary outcome parameters included ΔCBV, SpO2 and HR within the first 15 min after birth. Prespecified echocardiographic parameters included direction and amount of shunting via ductus arteriosus and foramen ovale, preload measures (superior vena cava flow and inferior vena cava size) and cardiac function measures (end-systolic right atrial size and area, end-diastolic plus end-systolic right ventricle size and area, tricuspid annular plane systolic excursion).

Although not prespecified in the study protocol, we further analyzed the results from the umbilical artery blood analysis.

### Randomization and blinding

Using a computer-generated randomization (www.randomizer.at) the included full-term neonates were randomly allocated to PBCC or control group at a 1:1 ratio. We used blocked randomization with a block size of 8. The attending clinical staff and midwife in charge of umbilical cord clamping was blinded until the scientific staff communicated to place the neonate on the mother's chest/abdomen with intact umbilical cord (PBCC group) or to clamp the cord (control group).

### Materials

NIRS measurements were performed with the NIRO 200 NX DP (Hamamatsu, Japan), and pulse oximetry was performed with the IntelliVue MP50 monitor (M1193A neonatal SpO2 wrap sensor, Koninkljke Philips; the Netherlands). For data analysis all parameters were stored automatically every second using a multichannel system “alpha-trace digital MM” (BEST Medical Systems; Austria). Echocardiography was performed by using LOGIQ S7 ultrasound system (GE Medical Systems, United States of America) with a 10 MHz probe.

### Near-infrared spectroscopy

NIRS is a continuous non-invasive method to measure changes in the concentration of oxygenated (ΔHbO2) and reduced hemoglobin (ΔHbR). Changes in total hemoglobin (ΔHbT) can be calculated by using the following equation:

(1) ΔHbT(μmol/l)=ΔHbO2+ΔHbR

The primary outcome parameter cTOI (%) was calculated according to the following equation:

(2) $$cTOI\;\left(\%\right)=\frac{\Delta HbO2}{\Delta HbT}*100$$

Moreover, ΔHbT (µmol/l) may be converted to changes in cerebral blood volume (ΔCBV) by using a previously described relationship (22). ΔCBV (ml/100 g brain) is calculated by the following equation, in which Hb represents the hemoglobin concentration (g/dl):

(3) $$\Delta CBV\;\left(ml/100\;g\;brain\right)\;=\Delta HbT*\frac{0.89}{Hb}$$

The NIRS device which was used for the present study relies on the “continuous wave spatially resolved” technique. This method does not allow measurements of absolute CBV, but of changes in CBV in proportion to HbT concentration, if the hemoglobin concentration and the large vessel to cerebral hematocrit ratio are presumed to be constant.

NIRS measurements were conducted over the whole study period of 15 min using a sample rate of 2 Hz. The interoptode distance was 4 cm, and a differential path length factor of 3.85 was chosen (23).

### Statistical methods

A linear mixed model with fixed effect for time and first-order autoregressive covariance structure was used for calculation of overall effects and differences between groups (PBCC vs. control) at each minute. The course of cTOI was analyzed starting with the second minute after birth until minute 15. The first minute after birth was not analyzed due to the high number of missing values. For the visualization of the courses of analyzed parameters estimated values according to the linear mixed model with 95% confidence intervals (95%CI) are shown. Continuous secondary outcome parameters such as ΔCBV, SpO2 and HR were analyzed in a comparable way. In each neonate, changes of mean ΔCBV values between each minute were calculated and then cumulated to reach the zero-line at minute 15. Minute 15 was used as reference value, as at that time point NIRS signal quality was most stable and reliable.

We derived individual hemoglobin levels for the conversion of ΔHbT to ΔCBV from the umbilical cord blood. For neonates with unsuccessful cord blood sampling, we used mean hemoglobin values of the respective study group to calculate ΔCBV. Demographic and clinical variables are presented as absolute and relative counts, mean and standard deviation (SD), or median and interquartile range (IQR), as appropriate. Comparisons of categorical baseline characteristics between neonates of the PBCC and control group were made using chi-square test and for continuous variables using t-test or Mann-Whitney U-test, as appropriate.

## Results

**Figure 1:**
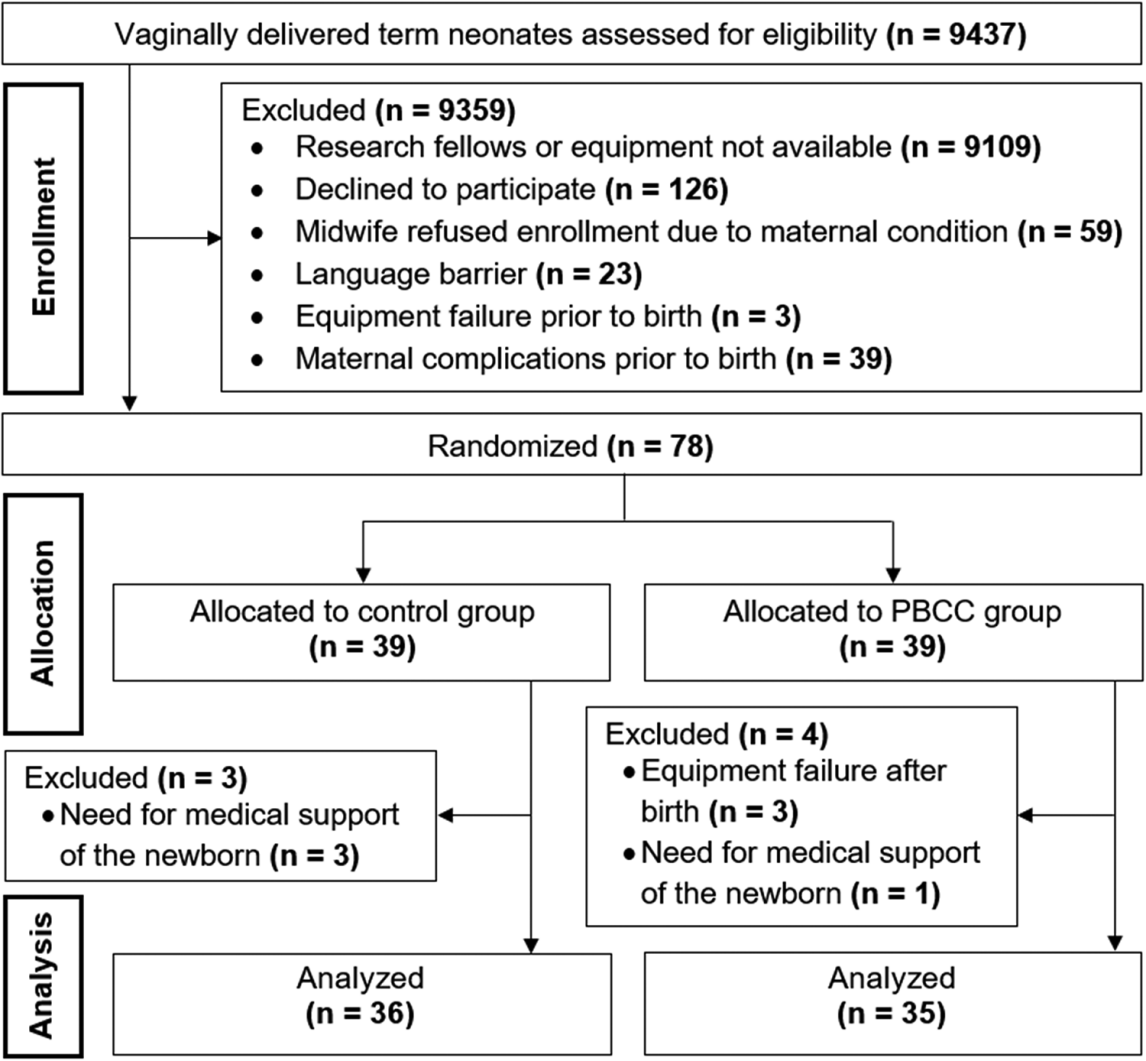
CONSORT diagram demonstrating enrollment, allocation and data analysis. PBCC, physiological-based cord clamping.

A total of 9,437 full-term neonates were vaginally delivered and assessed for eligibility in our center within the study period. The majority (*n* = 9359) needed to be excluded for several reasons (Figure 1), most of them because the research fellows or equipment were not available. Seventy-eight neonates were enrolled into the study and randomly assigned to the PBCC or control group. Seven patients were excluded due to requirement of medical support within the first 15 min after birth (*n* = 4) and equipment failure (*n* = 3). Finally, PBCC and control group consisted of 35 and 36 neonates, respectively (Figure 1).

The 71 included full-term neonates had a mean (SD) gestational age of 40.0 (1.0) weeks and a birth weight of 3,479 (424) g. The cord was clamped at a median (IQR) of 275 (197–345) and 58 (35–86) seconds in the PBCC and control group, respectively (*p* < 0.001). Demographic and clinical data of the neonates are shown in Table 1.

**Table 1:** Demographic and clinical characteristics of PBCC and control group. Data are presented as *n* (%), mean (SD), or median (IQR).

	PBCC group (*n* = 35)	control group (*n* = 36)	*p*-value
Gestational age (wk)	39^+6^ (1^+0^)	40^+0^ (1^+1^)	0.545
Birth weight (g)	3,493 (429)	3,465 (426)	0.782
Body length (cm)	51 (50–52)	52 (50–53)	0.419
Head circumference (cm)	35 (34–36)	35 (34–36)	0.599
Female sex	15 (43)	21 (58)	0.192
Apgar at 1 min	9 (9-9)	9 (9-9)	1.000
Apgar at 5 min	10 (10-10)	10 (10-10)	0.317
Apgar at 10 min	10 (10-10)	10 (10-10)	0.317
Time of cord clamping (s after birth)	***275*** **(*197***–***345)***	***58*** **(*35***–***86)***	***< 0.001*** ^a^
Time of first skin-to-skin contact (s after birth)	42 (24–71)	63 (32–90)	0.185
First displayed NIRS value (s after birth)	14 (0–89)	14 (0–96)	0.649
First displayed SpO2 value (s after birth)	121 (92–166)	123 (97–161)	0.956

^a^ *p <* 0.05; NIRS, near-infrared spectroscopy; PBCC, physiological-based cord clamping; SpO2, *peripheral* arterial oxygen saturation.

### Near-infrared spectroscopy monitoring

**Figure 2:**
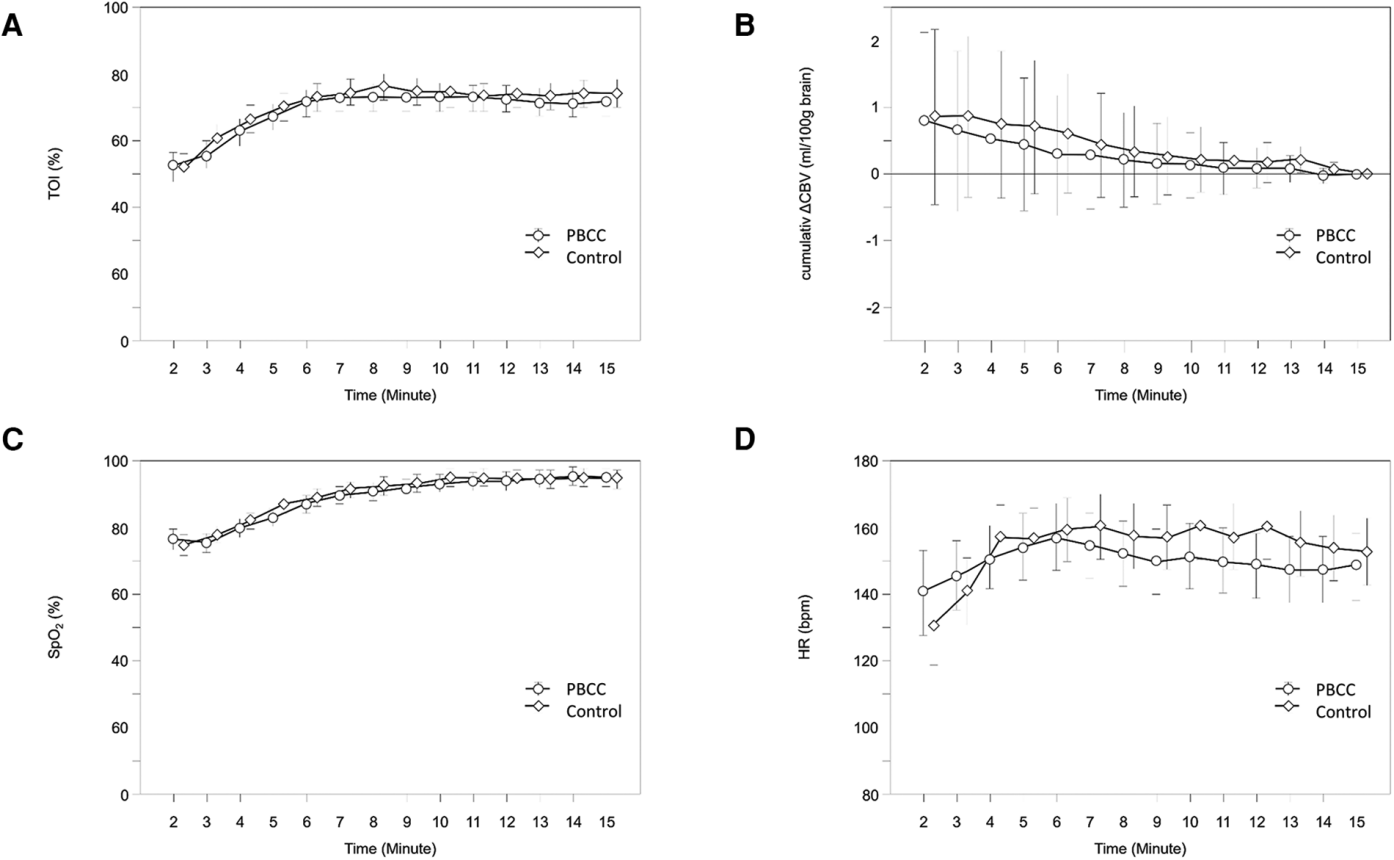
Courses of cTOI (**A**), ΔCBV (**B**), SpO2 (**C**) and HR (**D**) within the first 15 min after birth in PBCC (circles) and control group (diamonds); values are mean (95% CI). CBV, cerebral blood volume; cTOI, cerebral tissue oxygenation; HR, heart rate; SpO2, peripheral arterial oxygen saturation.

The first NIRS values were displayed in a median (IQR) of 35 (0–92) seconds after complete delivery of the neonate. In 28 neonates, the first values were already visible while only the neonate's head was delivered. In the whole study population cTOI increased significantly (*p* < 0.001) during immediate neonatal transition, and there were no significant differences in the courses of cTOI between the two groups (*p* = 0.319). CBV showed a strong tendency toward a decrease (*p* = 0.054), and there were no significant differences in the courses of ΔCBV (*p* = 0.814) between the two groups during the first 15 min after birth (Figure 2A,B).

### Pulse oximetry monitoring

The first pulse oximetry values were displayed in a median (IQR) of 121 (94–162) seconds after birth. SpO2 (*p* < 0.001) and HR (*p* = 0.006) increased significantly during the first 15 min after birth, with the largest increases within the first 10 and 5 min after birth, respectively. There were no significant differences in the courses of SpO2 (*p* = 0.322) and HR (*p* = 0.878) between the two groups during the first 15 min after birth (Figure 2C,D).

### Echocardiography

We stopped performing echocardiography after the inclusion of the 10th study participant following a controversial debate driven by the midwives (see limitations section). Echocardiography was performed in six out of the first ten participants. Complete data were available in only four participants. Due to the low number of cases a further analysis was omitted.

### Umbilical artery blood analysis

Umbilical artery blood analysis was performed in 66 of 71 (93%) neonates. There were significant differences in some of the umbilical artery blood parameters: Hemoglobin levels (*p* = 0.002) and pO2 levels were significantly higher (*p* = 0.002), whereas umbilical artery pH was significantly lower (*p* = 0.029) in the PBCC group compared to the control group. pCO2 showed a tendency toward higher levels in the PBCC group (*p* = 0.062) (Table 2).

**Table 2:** Umbilical artery blood parameters in PBCC and control group. Data are presented as mean (SD) or median (IQR).

	PBCC group (*n* = 35)	control group (*n* = 36)	*p*-value
pH	***7.24*** **(*7.19***–***7.27)***	***7.28*** **(*7.21***–***7.34)***	***0*** **.** ***029*** ^a^
pCO_2_ (mmHg)	53.0 (11.5)	47.4 (12.6)	0.062
pO_2_ (mmHg)	***30.9*** **(*25.6***–***40.1)***	***21.1*** **(*18.0***–***30.2)***	***0*** **.** ***002*** ^a^
Base excess (mmol/L)	−5.2 (2.8)	−5.2 (3.1)	0.992
HCO3^−^ (mmol/L)	18.8 (1.6)	19.0 (1.9)	0.642
Lactate (mmol/L)	5.2 (1.6)	5.4 (2.1)	0.670
Blood glucose (mg/dL)	77 (64–89)	87 (68–100)	0.098
Hemoglobin (g/dL)	***17.4*** **(*1.3)***	***16.4*** **(*1.4)***	***0*** **.** ***002*** ^a^

^a^ *p <* 0.05; HCO3^−^, hydrogen bicarbonate; PBCC, physiological-based cord clamping; pCO2, carbon dioxide partial pressure; pO2, oxygen partial pressure.

## Discussion

To the best of our knowledge, this randomized controlled trial is the first to compare different cord clamping strategies in a group of vaginally delivered healthy full-term neonates using NIRS technology for detailed evaluation of cTOI and ΔCBV during immediate neonatal transition (first 15 min after birth). In the intervention group we performed a physiological-based approach of cord clamping after the onset of stable regular breathing (PBCC group), while in the control group we performed a time-based approach of CC according to our standard procedure (control group). In contrast to our hypotheses, there were no significant group differences in the primary outcome parameter cTOI or in the secondary outcome parameters ΔCBV, SpO2 and HR. However, we found significant differences in some umbilical artery blood parameters including hemoglobin, pO2 and pH.

Experimental data demonstrated improved cerebral tissue oxygenation after PBCC in non-vigorous ventilated animals (11). In our study population of healthy vaginally delivered full-term neonates who were spontaneously breathing we could not observe such an effect. As previous clinical trials on PBCC and cerebral oxygenation are not available, we are unable to compare our results to such data. However, clinical trials on DCC indicated increased blood volume (7, 24), hematocrit (25–28), hemoglobin (1, 25, 26, 28) and iron stores (1, 25, 26), and improved cardiovascular function after birth particularly for premature infants (29, 30) which may increase oxygen delivery to the tissues. Nevertheless, there is only one study demonstrating improved cerebral tissue oxygenation in preterm neonates after DCC at 4 and 24 h after birth (27), whereas in other studies this effect was not seen (31, 32). In contrast, during immediate neonatal transition a two-center observational study demonstrated lower cerebral tissue oxygenation in preterm neonates after DCC (33). A trial on peripheral tissue oxygenation in term infants after DCC did not find changes in calf blood flow, oxygen delivery, oxygen consumption or fractional oxygen extraction (28). However, in the DCC studies the cord was clamped late irrespective of breathing/ventilation onset and establishment of lung aeration. Thus, further trials are warranted to elucidate the potential impact of PBCC on cerebral oxygenation. In the present trial we did not find improved cTOI following a potential increase of oxygen delivery, which probably may be explained by the fact that we investigated healthy full-term neonates who were spontaneously breathing and vigorous at birth, and demonstrated an uncomplicated neonatal transition after vaginal delivery. Therefore, the group differences might be too small for demonstrating a significant effect of this physiological-based approach. The above mentioned animal experimental studies (10, 11) suggest that PBCC is most appropriate in non-vigorous neonates in which lung aeration was established by positive pressure ventilation, and these neonates would be the ones most likely to benefit from PBCC.

The present study used NIRS technology for the evaluation of ΔCBV during the immediate neonatal transition in full-term neonates after vaginal delivery. Up to date there have been published reports on CBV during immediate neonatal transition in healthy full-term neonates (34, 35), in preterm and full-term neonates with and without respiratory support (36) and in preterm neonates receiving sustained lung inflations (37). All these studies showed a decrease of CBV during the first 15 min. The CBV drop most likely resulted from a steep pO2 increase after birth, triggering cerebral vasoconstriction (38). In our study population, CBV showed a strong tendency to decrease, which is in accordance with the previously published studies. Following the literature, we had hypothesized an increase of cTOI following PBCC, and thus a more pronounced decrease in CBV. In contrast to our hypotheses, there were no differences in the courses of cTOI (and SpO2), and ΔCBV in the present study. These observations are in accordance with an older study in preterm neonates with DCC or ECC, showing no differences in CBV at the postnatal age of 4, 24 and 72 h (27).

In the present study we further did not find differences in the routine parameters SpO2 and HR between groups. Mean SpO2 levels were close to 80% at 2 min, whereas the traditional Dawson percentiles were around 65% at 2 min (39). The recently published percentile charts by Badurdeen et al. (17) using data from 295 neonates born at ≥35 + 0 weeks of gestation who had DCC ≥ 2 min without respiratory support after birth showed also relatively lower SpO2 values (close to 70% at 2 min). Thus, to show any difference between the groups SpO2 would need to be >90% at 2 min in the PBCC group, which is not physiological in any group of neonates. We can only speculate on the relatively high SpO2 values in our study population, but reasons may include deferred cord clamping, gestational age (full-term neonates), tactile stimulation, vaginal birth, and a selection bias, since neonates in need for respiratory support were transferred to the resuscitation table and were excluded from the trial. In both groups mean HR was within the normal ranges of previously published percentiles (17, 40).

The umbilical arterial blood gas analyzes revealed significantly higher hemoglobin levels in the PBCC group (*p* = 0.002). It has been demonstrated that DCC resulted in higher hemoglobin levels in neonates at 24 to 48 h of age and during infancy (1, 25, 26, 28), emphasizing a similar effect of PBCC. However, it is remarkable to find those group differences already in the umbilical arterial blood, since changes in hemoglobin levels may be mainly dependent on fluid shift between the intravascular and interstitial compartment (transendothelial plasma leakage) which is supposed to be a rather slow process. In contrast to our study, other authors did not demonstrate group differences or reported on higher umbilical arterial hemoglobin levels after ECC vs. DCC although these results were not discussed in the Cochrane review or in the original studies reporting this outcomes (1). Current data do not allow a sufficient explanation for the findings.

Moreover, we found significantly higher levels of umbilical artery pO2 (*p* = 0.002) in the PBCC group, which is in accordance with two studies demonstrating that DCC (45–120 s) was associated with an increase in arterial pO2 (41, 42). At birth mixed blood is preferentially shunted right-to-left via ductus arteriosus into the descending aorta. After aeration of the lungs and decrease of pulmonary vascular resistance fetal circulation switches to postnatal conditions resulting in an increase in left ventricular output and oxygen delivery to the tissues. Therefore, the higher umbilical artery pO2 levels in the PBCC group may easily be explained by the already improved arterial oxygenation in the neonate at the deferred time point of CC.

pCO2 showed a tendency towards higher levels in the PBCC group (*p* = 0.063), while the umbilical artery pH values were significantly lower in the PBCC group (*p* = 0.031). We assume that the pH differences were mainly caused by blood gas differences rather than by metabolic factors, which is supported by similar lactate and hydrogen bicarbonate levels in both groups. A recent review on the effect of DCC (up to 120 s) on umbilical artery blood parameters in full-term neonates showed either no or only a small effect on cord blood acid-base balance (43). In accordance to our results two further studies (41, 44) reported decreased pH values and increased pCO2 levels after DCC. However, in contrast to our findings, they also found decreased hydrogen bicarbonate levels and increases in base deficit and lactate levels, demonstrating a shift in the acid-base status towards mixed acidosis. A recent observational study showed a shift towards metabolic acidosis only without changes in pCO2 in full-term neonates after vaginal birth (45). The reasons for these contradictory results remain unclear, different definitions of ECC and DCC and different time points of blood sampling might be possible explanations. However, it has to be noted that all of the umbilical blood parameters in our study population were within normal ranges. Therefore, the demonstrated differences most likely are clinically irrelevant.

Another influencing factor on the umbilical blood parameters might be the time points of umbilical arterial blood sampling and analyses, which unfortunately were not assessed for this study. Delayed blood sampling from the umbilical cord is known to result in an increase in pCO2 and a subsequent fall in pH following glycolysis as part of an ongoing cell metabolism (46, 47). In the PBCC group, blood sampling was potentially deferred compared to control group which would explain our pCO2 and pH findings.

### Limitations and strengths

All included neonates were full-term with a high likelihood of good adaptation after birth without requirement for respiratory support and were vaginally delivered. Thus, we selected a group of neonates in which adverse outcomes are unlikely to occur, which might be a reason why we couldn't investigate benefits following PBCC. The present study does not cover preterm neonates, neonates after Caesarean section, neonates receiving respiratory support or neonates with adverse conditions such as asphyxia or neonatal sepsis, which are factors that potentially affect the impact of PBCC.

The routine of CC slowly changed to longer CC times at our center, since obstetricians and midwives became increasingly aware of the potential benefits of DCC during the study period. This explains the difference between the expected CC time of 30–40 s in the control group and the actual values of 58 (35–86) seconds. This relatively long CC time in the control group might be a reason for diminishing or reducing differences between the groups.

Lung aeration could not be objectively evaluated by quantitative capnography or respiratory function monitoring, since we investigated spontaneously breathing healthy full-term neonates after vaginal birth with uncomplicated neonatal transition. The clinical assessment of the onset of stable regular breathing by the attending clinician might not adequately reflect the time point of establishment of optimal lung aeration, and pulmonary blood flow could not be assessed.

Since only healthy full-term born neonates were included, no blood samples of the neonates were collected for research purposes only due to ethical concerns. Hence, we cannot provide data regarding hemoglobin, hematocrit or bilirubin levels at birth or in the further course. Moreover, pO2 or pCO2 values after CC were not available, which might have been advantageous for the interpretation of the CBV results.

According to the study protocol, we planned to perform an echocardiography between 15 and 30 min after birth. Due to concerns of midwives and parents regarding disturbance of early skin-to-skin contact between mother and neonate following echocardiography, a controversial debate primarily driven by the midwives arose. To not endanger the continuation of the study, we decided to stop performing echocardiography after the 10th study participant despite positive approval of the ethics committee.

Although the targeted study population was 78 neonates, data from only 71 neonates could be analyzed as seven of them needed to be excluded for different reasons. Albeit, according to the study sample size calculation, it was required to include 35 neonates to each arm of the study, which could be finally achieved. This reiterates the importance of the predefined 10% fallout rate that was used in the sample calculation.

### Conclusion

In a group of healthy full-term neonates, there were no significant differences in the course of cTOI as well as ΔCBV, SpO_2_ and HR during the first 15 min after birth, irrespective of whether they received CC after the onset of stable regular breathing (intervention group) or within 1 min (control group). Thus, deferring CC ≥ 1 min following a physiological-based approach offers no benefits regarding cerebral tissue oxygenation and perfusion after uncomplicated vaginal delivery compared to a time-based CC approach. Further studies are needed investigating larger populations including neonates with adverse conditions such as prematurity, asphyxia, sepsis etc.

## Data Availability

The original contributions presented in the study are included in the article/Supplementary Material, further inquiries can be directed to the corresponding author/s.
